# Kynurenine and Tetrahydrobiopterin Pathways Crosstalk in Pain Hypersensitivity

**DOI:** 10.3389/fnins.2020.00620

**Published:** 2020-06-24

**Authors:** Ananda Staats Pires, Vanessa X. Tan, Benjamin Heng, Gilles J. Guillemin, Alexandra Latini

**Affiliations:** ^1^Neuroinflammation Group, Department of Biomedical Sciences, Centre for Motor Neuron Disease Research, Faculty of Medicine, Health and Human Sciences, Macquarie University, Sydney, NSW, Australia; ^2^Laboratório de Bioenergética e Estresse Oxidativo, Departamento de Bioquímica, Centro de Ciências Biológicas, Universidade Federal de Santa Catarina, Florianópolis, Brazil

**Keywords:** chronic pain, neuropathic pain, inflammatory pain, neuroinflammation, kynurenine, tetrahydrobiopterin, xanthurenic acid, central sensitization

## Abstract

Despite the identification of molecular mechanisms associated with pain persistence, no significant therapeutic improvements have been made. Advances in the understanding of the molecular mechanisms that induce pain hypersensitivity will allow the development of novel, effective, and safe therapies for chronic pain. Various pro-inflammatory cytokines are known to be increased during chronic pain, leading to sustained inflammation in the peripheral and central nervous systems. The pro-inflammatory environment activates additional metabolic routes, including the kynurenine (KYN) and tetrahydrobiopterin (BH4) pathways, which generate bioactive soluble metabolites with the potential to modulate neuropathic and inflammatory pain sensitivity. Inflammation-induced upregulation of indoleamine 2,3-dioxygenase 1 (IDO1) and guanosine triphosphate cyclohydrolase I (GTPCH), both rate-limiting enzymes of KYN and BH4 biosynthesis, respectively, have been identified in experimental chronic pain models as well in biological samples from patients affected by chronic pain. Inflammatory inducible KYN and BH4 pathways upregulation is characterized by increase in pronociceptive compounds, such as quinolinic acid (QUIN) and BH4, in addition to inflammatory mediators such as interferon gamma (IFN-γ) and tumor necrosis factor alpha (TNF-α). As expected, the pharmacologic and genetic experimental manipulation of both pathways confers analgesia. Many metabolic intermediates of these two pathways such as BH4, are known to sustain pain, while others, like xanthurenic acid (XA; a KYN pathway metabolite) have been recently shown to be an inhibitor of BH4 synthesis, opening a new avenue to treat chronic pain. This review will focus on the KYN/BH4 crosstalk in chronic pain and the potential modulation of these metabolic pathways that could induce analgesia without dependence or abuse liability.

## Introduction

The immune and pain-signaling systems are evolutionarily designed to protect the organism by acutely responding to danger (Woolf and Ma, 2007; Woolf, 2010; O’neill et al., 2016). Typically, stimuli that activate both systems elicit inflammation and pain that are adaptive response to overcome the threat, increasing the life-time reproductive success (Beringer and Miossec, 2019). However, many injuries and diseases may perpetuate maladaptive inflammatory reactions, in which pro-inflammatory mediators persistently activate and sensitize neurons at different levels of the nociceptive pathway (Woolf and Costigan, 1999; Costigan et al., 2009a, 2010; Oikawa et al., 2019). These long-lasting sensory changes known as chronic pain, represents a major unmet clinical need (for a review see Woolf and Salter, 2000). This is a significant problem due to the high incidence worldwide (Rice et al., 2016) and lack of effective, specific and safe therapies (Varrassi et al., 2010; Dart et al., 2015; Jones, 2017).

Neuroinflammation is characterized by the infiltration of peripheral immune cells, activation of glial cells and production of inflammatory mediators in the peripheral and central nervous systems (PNS; CNS). This contributes to generate a peripheral and central sensitization that causes long-term pain (Kawasaki et al., 2008; Gao and Ji, 2010). Therefore, targeting these neuroinflammatory processes and molecules may result in an effective analgesic treatment for chronic pain.

## Chronic Pain

Chronic pain is a dysfunctional process defined by longstanding pain sensations of more than three months (IASP, 2019; Treede et al., 2019). Chronic pain is a major health problem worldwide that negatively impacts on the quality of life of the affected individuals, and represents a huge health public costs in both developed and emerging countries (Goren et al., 2014). Approximately 20% of the adult population in the United States (Dahlhamer et al., 2018), Canada (Shupler et al., 2019), and Europe (Breivik et al., 2006) are affected by chronic pain. Similar prevalence is observed in Australia, about 15% (Miller et al., 2017), and higher in countries like Brazil and Japan, with a prevalence of around 40% (Inoue et al., 2015; De Souza et al., 2017). Chronic pain is a major health burden to the society, with annual costs over $635 billion per year in the United States alone (Gereau et al., 2014). This exceeds the combined costs of common chronic conditions including, cancer, heart disease, and diabetes (Gereau et al., 2014). The global high incidence of chronic pain is aggravated by the lack of effective and safe treatments; in particular, the development of side effects such as addiction and the risk overdose leading to death (NIDA, 2020).

There are numerous classes of drugs used to treat pain, including serotonin reuptake inhibitors, non-steroidal anti-inflammatory drugs (NSAIDs), and opioids (Nalamachu, 2013). Each of these drugs are associated with different adverse events impacting the gastrointestinal, cardiovascular, and renal systems, and their efficacy against chronic pain is controversial (Macario and Lipman, 2001; Brueggemann et al., 2010; Skljarevski et al., 2012; Chou et al., 2014). A recent meta-analysis showed that opioids, the most efficient class of drug against acute pain, provided only minor improvements for people dealing with chronic pain caused by conditions other than cancer (Busse et al., 2018). Moreover, the repeated use of opioids is strongly associated with addiction and risk of death (NIDA, 2020). Therefore, more research is urgently needed to develop pain medications with higher efficacy and safety.

The exact mechanism driving pain persistence is poorly understood. Chronic pain conditions are likely to have distinctive underlying mechanisms that ultimately alter the long-term nociceptive signaling in patients. Indeed, a key and common feature for all chronic pain conditions is a long-term neuronal plasticity in pain-signaling circuits that result in increased neuronal responsiveness to their normal input and/or recruitment of a response to subthreshold inputs (for a review see Woolf and Salter, 2000). The pain-induced neuronal plasticity involves the sensitization of sensory neurons in different anatomical locations along of the PNS and CNS (Woolf, 1983; Latremoliere and Woolf, 2009).

## Nociceptive Signaling Pathway and Pain Hypersensitivity

The peripheral primary sensory neurons from the pain-signaling pathway are activated by different noxious stimuli, including thermal, mechanical, or chemical stimuli that have potential or are currently damaging tissue. These specialized primary sensory cells are pseudo-unipolar neurons with the soma anatomically located in the dorsal root ganglia (DRG) and in the trigeminal ganglia (Kandel, 2013). The peripheral terminals of nociceptors are equipped with a range of receptors that transduce noxious stimuli into action potentials, which are then transmitted through the nervous systems. All primary sensory nociceptors, through their central terminals, make synaptic connections with second-order neurons in the spinal cord (for a review see Woolf and Ma, 2007). Some subsets of spinal dorsal horn neurons project axons and transmit pain messages to higher brain centers, including the reticular formation, thalamus, amygdala, and finally the cerebral cortex. These brain regions are associated with autonomic, hormonal, emotional and cognitive aspects of pain, including the perception and consciousness of pain (Mobbs et al., 2009). The neural activity along the pain transmission pathway is inhibited or amplified by ascending and descending neural circuits (for a revision see Woolf, 2018). This modulation allows a wide range of factors to modulate pain sensation and perception, including psychosocial and environmental factors (for a review see Chayadi and Mcconnell, 2019).

## Neuroinflammation in Chronic Pain

**FIGURE 1:**
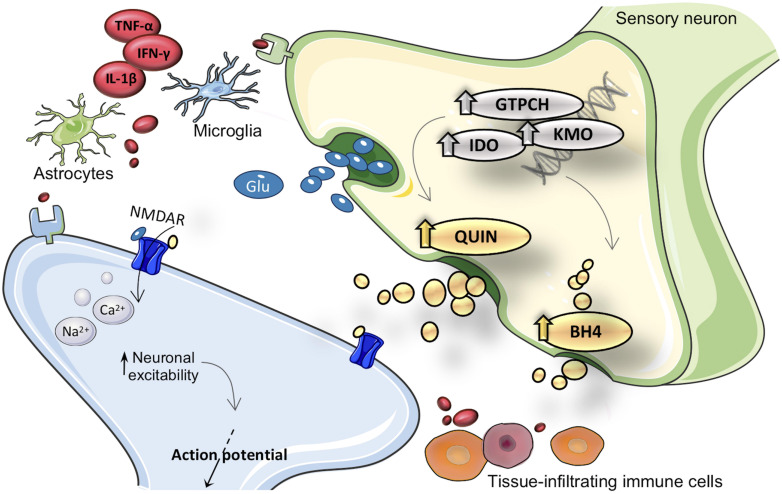
Merger of kynurenine (KYN) and tetrahydrobiopterin (BH4) pathways into inflammation cascade involved in pain hypersensitivity. After inflammatory activation, expression and activity from GTPCH (guanosine triphosphate cyclohydrolase I), IDO1 (indoleamine 2,3-dioxygenase), and KMO (kynurenine 3-monooxygenase) enzymes are enhanced in brain and immune cells. Inflammatory inducible KYN/BH4 pathways upregulation is characterized by increased nociceptive compounds, such as QUIN (quinolinic acid) and BH4. As a consequence, neurons undergo profound changes in activity, which eventually result in exacerbated pain sensations. The neuroactive compound QUIN is NMDAR (N-methyl-d-aspartate receptor) agonist it is proposed to exacerbate hypersensitivity throughout modulation of this receptor. The exact molecular mechanism by which BH4 induces pronociceptive effects is not fully elucidated. TNF-α, tumor necrosis factor alpha; IL-1β, interleukin-1beta; IFN-γ, interferon gamma; Glu, glutamate.

Growing evidence suggests that persistent inflammation within the PNS and CNS is a factor that drives self-perpetuation and pathologic plasticity changes in sensory neurons, sustaining the chronicity of pain (Kawasaki et al., 2008; Gao and Ji, 2010). Injuries and diseases that directly or indirectly affect the PNS and CNS can elicit neuroinflammatory responses that include activation of resident immune cells, changes in capillary permeability and infiltration of peripheral blood cells (for a review see Zhuang et al., 2005). It has been demonstrated that during inflammation peripheral leukocytes (including neutrophils, monocytes/macrophages and T cells) are able to infiltrate the PNS and CNS, leading to overproduction of a variety of pro-inflammatory cytokines, chemokines, and other pain-related mediators (Costigan et al., 2009b; Kigerl et al., 2009). The neuroinflammatory response is further amplified by the activation of resident glial cells, including microglia and astrocytes. Once activated, these cells undergo hypertrophic changes and increase the release of glial mediators, including numerous trophic factors, chemokines and proinflammatory cytokines that can modulate pain sensitivity (Rojewska et al., 2014b; Figure 1).

While acute neuroinflammation can produce transient peripheral and central sensitization, permanent or repeated neuroinflammation is associated with a long-lasting and even permanent sensitization (Christianson et al., 2011). The literature supports the association between neuroinflammation and various chronic pain conditions, such as neuropathic pain triggered by diabetes, nerve and spinal cord injury, inflammatory pain caused by arthritis, inflammatory bowel disease, cancer related pain, complex regional pain syndrome, and pain caused by drug therapy (Sweitzer et al., 2002; Chua et al., 2019). As an example, a study on *post mortem* spinal cord samples from human immunodeficiency virus (HIV)-infected patients with neuropathic pain showed increased glial activation and increased inflammatory cytokine levels (Shi et al., 2012).

The exact mechanisms by which neuroinflammation favors the transition from acute pain to persistent pain is still poorly defined. This lack of understanding of the basic mechanisms of pain perpetuation is reflected in the limited efficacy of anti-inflammatory drugs, in addition to the significant side effects (Enthoven et al., 2016). Therefore, new avenues need to be explored in order to manage this unmet clinical condition. In this paradigm, emerging mediators related to inflammation-enhanced metabolic pathways, *i.e.*, tetrahydrobiopterin (BH4) and quinolinic acid (QUIN; a KYN pathway metabolite) have been proposed to favor pain hypersensitivity (Latremoliere et al., 2015; Laumet et al., 2017). The innate immune system once activated elicits the synthesis of pro-inflammatory mediators in order to coordinate the inflammatory response. Many of these mediators can transcriptionally upregulate the expression of inducible enzymes, activating the pathological production of BH4 and QUIN (for a review see Guillemin, 2012; Ghisoni et al., 2015a; Figure 1).

## The Biosynthesis of BH4

BH4 is traditionally known as an essential cofactor for the catalytic activity of phenylalanine hydroxylase, tyrosine-3-hydroxylase, tryptophan-5-hydroxylase, alkylglycerol monooxygenase, and all nitric oxide synthase isoforms (Thöny et al., 2000). As a consequence, BH4 is crucial for hydroxylation of the aromatic amino acids, resulting in the catabolism of phenylalanine and the synthesis of the catecholaminergic neurotransmitters dopamine and serotonin. BH4 is also mandatory for the cleavage of ether lipids as well as the biosynthesis of nitric oxide (Thöny et al., 2000; Werner et al., 2011). Recently, our group has uncovered other fundamental physiological roles for basal BH4 levels, as having antioxidant and anti-inflammatory properties, and being a mitochondrial activator as well as a memory enhancer (Ghisoni et al., 2015a, b, 2016; De Paula Martins et al., 2018; Latini et al., 2018). We also described that exacerbated production of BH4 is pathogenic, causing pain, increasing the aggressiveness of the immune system and the progression of the symptoms of chronic diseases, including, chronic pain, asthma, multiple sclerosis, ulcerative colitis, rheumatoid arthritis, and cognitive impairment (Latremoliere et al., 2015; Cronin et al., 2018; Fujita et al., 2019).

**FIGURE 2:**
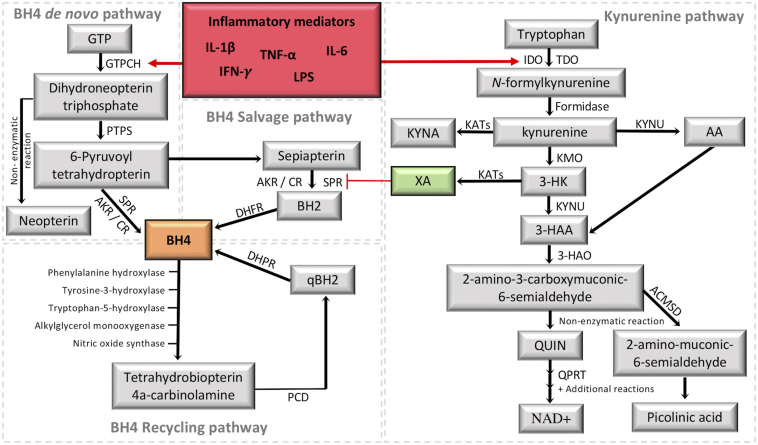
Crosstalk between the tetrahydrobiopterin (BH4) and kynurenine (KYN) pathways. It is highlighted in red the activation of the two metabolic pathways under inflammation. It is highlighted in green the KYN intermediate xanthurenic acid (XA), which recently was demonstrated to be an inhibitor of sepiapterina reductase (SPR) (Haruki et al., 2016). The formation of BH4 (highlighted in orange) by the *de novo* pathway involves the catalytic activity of GTPCH (guanosine triphosphate cyclohydrolase I), PTPS (followed by 6-pyruvoyl tetrahydropterin synthase) and SPR (sepiapterin reductase). SPR deficiencies may be overcome in target tissues by unspecific reductases of the salvage pathway, including aldoketo and carbonyl reductases (AKR; CR) (Hirakawa et al., 2009; Werner et al., 2011), which transform 6-pyruvoyl-tetrahydropterin into sepiapterin and BH2 (7,8-dihydrobiopterin), then the final reduction back to BH4 is performed by DHFR (dihydrofolate reductase). The recycling pathway maintains high levels of BH4 in the liver, where it is mainly used to metabolize phenylalanine. After BH4 oxidation, PCD (Pterin 4a-carbinolamine dehydratase) forms qBH2 (quinonoid dihydrobiopterin) to be reduced back to BH4 by DHPR (dihydropteridine reductase). The KYN pathway transforms tryptophan into a series of intermediates with different properties by combining the activity of several enzymes in different cells/tissues. The central intermediate KYN allows the production of XA, which inhibits SPR activity in the BH4 pathway. GTP, guanosine triphosphate; IDO1, indoleamine 2,3-dioxygenase; TDO, tryptophan 2,3-dioxygenase; KYNU, kynureninase; KYNA, kynurenic acid; KATs, kynurenine aminotransferases; KMO, kynurenine 3-monooxygenase; 3-HK, 3-hydroxykynurenine; AA, anthranilic acid; 3-HAA, 3-hydroxyanthranilic acid; 3-HAO, 3-hydroxyanthranilic acid 3,4-dioxygenase; QUIN, quinolinic acid; QPRT, quinolinate phosphoribosyltransferase; NAD^+^, nicotinamide adenine dinucleotide; ACMSD, 2-amino-3-carboxymuconate-6-semialdehyde decarboxylase; IFN-γ, interferon gamma, TNF-α, tumor necrosis factor alpha; IL-1β, interleukin 1beta; IL-6, interleukin-6; LPS, lipopolysaccharides.

Physiological basal levels of BH4 are at tightly controlled concentrations, requiring therefore, a tuned regulation of BH4 synthesis. Three metabolic pathways, namely *de novo* synthesis, recycling, and salvage pathways cooperate to maintain appropriate intracellular levels of BH4 (Figure 2). The *de novo* pathway generates BH4 from guanosine triphosphate (GTP) through a three-step enzymatic cascade starting with the rate-limiting enzyme guanosine triphosphate cyclohydrolase I (GTPCH), followed by 6-pyruvoyl tetrahydropterin synthase (PTPS) and sepiapterin reductase (SPR) (for a review see Ghisoni et al., 2015a). Alternative to *de novo* synthesis, intracellular BH4 levels can be produced via the salvage pathway using sepiapterin and 7,8-dihydrobiopterin as intermediates. Although this pathway is not fully understood, SPR and dihydrofolate reductase (DHFR) appear to be essential enzymes to maintain BH4 levels without consuming high-energy phosphate containing compounds (Werner et al., 2011). In addition, the catalytic activity of SPR can also be performed by non-specific enzymes, the aldoketo and carbonyl reductases (Hirakawa et al., 2009; Werner et al., 2011). Finally, the recycling pathway represents a mechanism that preserves energy and generates large quantities of pterin in high-BH4 demanding organs (e.g., hepatic metabolism of aromatic amino acids). After BH4 participates as a mandatory enzymatic cofactor, the unstable intermediate 4a-hydroxy-tetrahydrobiopterin is formed and undergoes a dehydration leading to the formation of quinonoid dihydrobiopterin, which is reduced back to BH4 by dihydropteridine reductase (Thöny et al., 2000; Longo, 2009).

Increased levels of BH4 are expected under cellular stress and requires the production of new blocks of BH4. GTPCH, the rate-limiting enzyme of the *de novo* BH4 pathway, is an inducible enzyme, having its expression controlled by pro-inflammatory mediators, such as interferon gamma (IFN-γ), tumor necrosis factor alpha (TNF-α), interleukin-1beta (IL-1β), and lipopolysaccharides (LPS) (Werner et al., 1990). During the pro-inflammatory response, expression of *GCH1* (which codes for GTPCH), and GTPCH activity are markedly increased, while the downstream enzymes, PTPS and SPR, are only slightly augmented, resulting in PTPS as the rate-limiting enzyme of the BH4 *de novo* pathway during inflammation. Consequently, this pseudometabolic blockage favors the accumulation of the PTPS substrate, which non-enzymatically will be transformed in neopterin, a well-established and sensitive biomarker for immune system activation, and for the activation of the *de novo* BH4 pathway (for a review see Ghisoni et al., 2015a; Figure 2).

## BH4 and Chronic Pain

The first human validation linking chronic pain to the BH4 metabolism comes from the identification of single nucleotide polymorphisms in the *GCH1* loci, which correlated with reduced experimental and clinical persistent pain sensitivity (Tegeder et al., 2006, 2008). Human homozygous haplotypes were associated with decreased upregulation of *GCH1* upon inflammatory stimulation, but without full loss of function of GTPCH, which would preserve baseline BH4 concentrations (Campbell et al., 2009; Doehring et al., 2009; Dabo et al., 2010; Kim et al., 2010, 2013). Indeed, it was demonstrated that this human protective-pain haplotype only influences nociceptive thresholds after pain sensitization (Tegeder et al., 2008). In addition, individuals carrying the homozygous from of the haplotype are less sensitive to persistent leg pain after discectomy or persistent pain in fibromyalgia (Tegeder et al., 2008; Kim et al., 2010, 2013). However, other studies demonstrated that carrying this human homozygous polymorphism does not protect from pancreatic pain, post dental surgery or post mastectomy pains, suggesting that the association between *GCH1* and pain may be disease- or tissue-specific (Kim and Dionne, 2007; Lazarev et al., 2008; Holliday et al., 2009; Hickey et al., 2011).

In rodent models, the genetic ablation of *Gch1* in DRGs generated evidence supporting the contribution of excessive levels of BH4 in chronic pain hypersensitivity (Kim et al., 2009; Latremoliere et al., 2015). DRG *Gch1* null rats showed decreased mechanical pain hypersensitivity and microglial activation in the dorsal horn 14 days following spared nerve injury (SNI) (Kim et al., 2009). Similarly, the absence of *Gch1* in the DRG of mice prevented excessive BH4 production in sensory neurons and mechanical pain hypersensitivity induced by nerve injury following 21 days of SNI and chronic constriction injury (CCI) as measured by the normalization of the mechanical threshold for pain (Latremoliere et al., 2015).

Expression and functional profiling in rodents has shown that enhanced BH4 biosynthetic enzymes transcription and activity in sensory neurons and immune cells lead to increased BH4 levels, which results in greater chronic pain hypersensitivity (Tegeder et al., 2006; Kim et al., 2009; Latremoliere et al., 2015; Cronin et al., 2018). For example, *Gch1* and *Spr* were upregulated up to sixfold 21 days after SNI, indicating that elevated transcription for BH4 biosynthesis persists for some time after injury (Tegeder et al., 2006; Kim et al., 2009; Latremoliere et al., 2015). In parallel with persistent mechanical hypersensitivity, BH4 levels are increased in rat sensory neurons in response to both axonal injury and peripheral inflammation induced by SNI and intraplantar complete Freund’s adjuvant (CFA) injection, respectively (Tegeder et al., 2006). Furthermore, enhanced *GCH1* transcription and increased BH4 levels were identified not only in injured sensory neurons, but also in leukocytes that infiltrated the tissue after SNI in mice, which underline the contribution of the immune system in the persistence of hypersensitivity induced by BH4 overproduction (Latremoliere et al., 2015). Collectively, the enhanced expression of the transcripts for the BH4 biosynthetic enzymes and increased BH4 levels correlate with the persistence of mechanical and cold hypersensitivity in rat and mice models for inflammatory and neuropathic pain, reinforcing the contribution of excessive levels of BH4 with chronic pain hypersensitivity (Tegeder et al., 2006; Latremoliere et al., 2015; Fujita et al., 2019).

### Inhibition of Pathological BH4 Levels as a Novel Pathway to Induce Analgesia

One of the most effective current pharmacological therapies for controlling certain types of pain is the use of opioids. However, chronic opioid use lacks safety, effectiveness and has a substantial liability for abuse and high risk of death from overdose (NIDA, 2020). Therefore, the inhibition of inflammation-triggered BH4 production may represent an innovative and non-addictive strategy for managing persistent pain.

Analgesia induced by the pharmacological inhibition of GTPCH activity was demonstrated in rodents subjected to SNI and CCI. The use of 2,4-diamino-6-hydroxypyrimidine (DAHP; a GTPCH inhibitor) reversed the mechanical and cold hypersensitivity induced by the nerve injury (Tegeder et al., 2006). Similarly, DAHP treatment reduced tumor-evoked microglial activation in the spinal cord and reduced cancer-induced systemic hyperalgesia in mice (Pickert et al., 2012). These initial data indicated that targeting the flux of this metabolic pathway could represent new horizons in the clinical management of chronic pain. However, since GTPCH activity is essential for BH4 production, any pharmacological approaches should aim to reduce exacerbated BH4 levels back to basal levels, without compromising its physiological roles on endothelial function and metabolisms of neurotransmitters, lipids and nitric oxide.

A yeast three hybrid screen revealed that a Food and Drug Administration (FDA)-approved anti-inflammatory compound, sulfasalazine (SSZ), is an inhibitor of the BH4 synthesizing enzyme SPR (Haruki et al., 2013). The use of SSZ in mice subjected to SNI showed reduced mechanical pain hypersensitivity, without compromising essential BH4-related functions (Latremoliere et al., 2015). However, the analgesia induced was mild, probably due to the limited bioavailability, low potency, and complex metabolism of this drug in the gut (Pieniaszek and Bates, 1979). Thus, using a structure-based design, our group has developed new more potent SPR inhibitors (SPRi), SPRi3 and QM385. These SPRi have been shown to induce potent analgesic effects in neuropathic and inflammatory pain models, without inducing tolerance or adverse effects (Latremoliere et al., 2015; Fujita et al., 2019). Either a single or repeated SPR inhibitor intraperitoneal injection alleviated intraplantar CFA injection-, SNI-, and CCI-induced pain hypersensitivity in mice, with a maximal activity 1 h after the administration (Latremoliere et al., 2015). The analgesic effect of these SPRi were also demonstrated in the chronic phase of the collagen antibody-induced arthritis (CAIA) model of inflammatory joint pain, in which a rapid onset of clinical signs of arthritis is followed by a persistent pain-related hypersensitivity syndrome lasting at least 55 days (Fujita et al., 2019). We also demonstrated that SPRi reduced pain scores in mice submitted to a colitis experimental model, and that this effect was in part due to the absolute requirement of T cells for BH4 in order to expand and infiltrate tissues (Cronin et al., 2018). Another FDA-approved drug, Tranilast – an anti-allergic agent, was also demonstrated to inhibit SPR in protein- and cell-based assays (Moore et al., 2019) and to reduce pelvic pain caused by endometriosis in a clinical study (Honda et al., 2013).

Table 1 summarizes the half maximal inhibitory concentration (IC_50_) values of key SPRi, including the natural SPR inhibitors (e.g., N-acetyl-serotonin), the synthetic and FDA-approved compounds.

**TABLE 1:** Half maximal inhibitory concentration (IC_50_) for the known sepiapterin reductase inhibitors (SPRi).

Compounds	IC_50_ value (μM)		
	*In vitro*		*In vivo*
	Protein-based assay	Cellular system	
**Natural SPRi**			
N-acetyl-serotonin	3.8^h^	∅	∅
	35^m^		
	1.2^r^		
	(Haruki et al., 2016)		
	11.61^h^		
	(Moore et al., 2019)		
Xanthurenic acid	0.15^h^	∅	∅
	0.053^m^		
	0.045^r^		
	(Haruki et al., 2016)		
**Synthetic SPRi**			
SPRi3	0.053^h^	0.45^a^	∅
	(Haruki et al., 2016)	Latremoliere et al., 2015	
QM385	∅	0.036^b^	∅
		0.074^c^	
		Cronin et al., 2018	
Q-1195	0.008^h^	∅	2.2^d^
	0.004^r^		
	Meyer et al., 2019		
			(Meyer et al., 2019)
**FDA-approved**			
Sulfasalazine	0.0070^h^	∅	∅
	0.0078^m^		
	0.043^r^		
	(Haruki et al., 2016)		
	0.023^h^		
	(Haruki et al., 2013)		
Tranilast	5.889^h^	∅	∅
	(Moore et al., 2019)		

*Values are mean of at least 2 independent experiments. ∅, not determined. h, human SPR; m, mouse SPR; r, rat SPR. a, mouse primary dorsal root ganglia cell culture. b, mouse splenocytes cell culture. c, human peripheral blood mononuclear cell culture. d, adult male Sprague-Dawley rats after spinal nerve injury. FDA, Food and Drug Administration.*

### Sepiapterin as a Biological Marker for the Analgesic Effects of SPRi

Sepiapterin is a metabolic intermediate of the BH4 salvage pathway. It does not accumulate intracellularly, but is found at increased concentrations in the biological fluids of patients affected by mutations in the SPR gene (Carducci et al., 2015). The chemical stability of sepiapterin and its accumulation upon genetic and/or pharmacological manipulation of SPR make this metabolite a sensitive and specific biological marker of SPR inhibition (Fujita et al., 2019). Indeed, a dose-dependent increase of sepiapterin levels in plasma and urine from rodents, and humans were observed after the administration of SPRi (SSZ, SPRi3, QM385, and Q1195 in rodents and SSZ in humans (Latremoliere et al., 2015; Fujita et al., 2019; Meyer et al., 2019). In a recent publication by our group, the urinary sepiapterin was established as a reliable biomarker of SPR inhibition with high sensitivity (70–85%) and specificity (82–88%) in both mice and human health volunteers after the administration of SPRi3 and SSZ, respectively (Fujita et al., 2019).

In an effort to associate changes of sepiapterin and BH4 with analgesic effects induced by SPRi, Table 2 shows the changes on sepiapterin and BH4 levels identified in biological samples after treatment with different SPRi and the correspondent analgesic effects in different experimental pain models.

**TABLE 2:** Analgesic effects, tetrahydrobiopterin (BH4) and sepiapterin changes for the main sepiapterin reductase inhibitors (SPRi).

SPRi	Analgesic effect	Experimental pain model	BH4 changes	Sepiapterin changes
N-acetyl-serotonin	⇓ Cold allodynia; ⇓ Mechanical allodynia (Tegeder et al., 2006)	Spared nerve injury neuropathic pain model	∅	∅
	⇓ Heat hyperalgesia (Tegeder et al., 2006)	Granulomatous skin inflammatory pain model (intra-plantar injection of CFA)	∅	∅
SPRi3	⇓ Heat hyperalgesia; ⇓ Mechanical allodynia (Fujita et al., 2019)	Collagen antibody-induced arthritis model	⇓ in urine	⇑ in urine
	⇓ Mechanical allodynia (Latremoliere et al., 2015)	Chronic constriction injury and spared nerve injury neuropathic pain models	⇓ in DRG, sciatic nerve and plasma	⇑ in DRG, sciatic nerve and plasma
	⇓ Heat hyperalgesia; No changes in mechanical allodynia (Latremoliere et al., 2015)	Granulomatous skin inflammatory pain model (intraplantar injection of CFA)	⇓ in plantar skin	∅
QM385	⇓ Heat hyperalgesia (Fujita et al., 2019)	Collagen antibody-induced arthritis model	∅	⇑ in plasma
Q-1195	No changes in mechanical allodynia (Meyer et al., 2019)	Spinal nerve ligation neuropathic pain model	⇓ in DRG	⇑ in plasma and DRG
Sulfasalazine	⇓ Mechanical allodynia (Latremoliere et al., 2015)	Spared nerve injury neuropathic pain model	∅	⇑ in plasma

*∅ not evaluated. CFA, complete Freund’s adjuvant; DRG, dorsal root ganglia.*

## The KYN Pathway Biosynthesis

The KYN pathway is active in a variety of different tissues, but more notably in the liver through the enzyme tryptophan 2,3-dioxygenase (TDO); and in cells of the immune and nervous systems (including neurons, microglia and astrocytes) by indoleamine 2,3-dioxygenase 1 (IDO1) (Moroni et al., 1988a). IDO1 is the rate-limiting enzyme of the pathway in immune cells, playing key roles in immune system activation and regulation (Guillemin et al., 2005). The most potent activator of IDO1 is IFN-γ (Werner-Felmayer et al., 1989), but this enzyme is also activated by other mediators such as LPS, amyloid peptides, cytotoxic T lymphocyte antigen-4 (CTLA4) and HIV proteins (Guillemin et al., 2003; Jones et al., 2015). Around 95% of the dietary tryptophan (Trp) is metabolized into the KYN pathway, which can follow three different metabolic routes, synthesizing the essential cofactor nicotinamide adenine dinucleotide (NAD^+^), kynurenic acid (KYNA), or xanthurenic acid (XA) (Beadle et al., 1947; Wolf, 1974; Figure 2).

Initially, Trp can be oxidized into the instable metabolite N-formyl-kynurenine by TDO or IDO1, to be further transformed into KYN, the central intermediate of the pathway, by formamidase. KYN can be metabolized into anthranilic acid or KYNA by kynureninase and kynurenine aminotransferases (KATs I, II, and III), respectively. Additionally, kynurenine 3-monooxygenase (KMO) can transform KYN into 3-hydroxykynurenine (3-HK) to produce 3-hydroxyanthranilic acid (3-HAA) by kynureninase. 3-HAA in turn, forms picolinic acid, QUIN and NAD^+^ through the action of additional enzymes. 3-HK can be also metabolized by KATs into XA (Figure 2).

## KYN Pathway and Chronic Pain

There is extensive evidence in literature that proinflammatory stimuli, mitochondrial dysfunction, oxidative stress, and the formation of neuroactive metabolites that can modulate glutamatergic receptors and neurotransmitter production are relevant to pain sensation (Moroni et al., 1988b; Turski et al., 1988). Several of the KYN pathway metabolites are neuroactive compounds able to regulate, for example, the activity of glutamatergic N−methyl−d−aspartate (NMDA) receptors inducing toxicity, favoring the excessive generation of reactive species (Stone and Perkins, 1981; Kessler et al., 1989), or compromising the activity of the energy metabolism by deficiencies in the synthesis of Trp-linked NAD^+^ (Massudi et al., 2012; Gomes et al., 2013; Zhu et al., 2015). For example, QUIN is a potent NMDA receptor agonist, which at nM levels induces excitotoxicity, mitochondrial damage, oxidative stress, destabilization of the cellular cytoskeleton, and disruption of autophagy, among other negative effects (Schwarcz et al., 1984; Guillemin, 2012). Thus, perturbations on the KYN pathway may favor the transition from acute to persistent pain by inducing these deleterious reactions. In this scenario, it is well established that glutamatergic neurotransmission is essential for peripheral (Pardutz et al., 2012) and central sensitization (Latremoliere and Woolf, 2009). Thus, the pharmacological antagonism of NMDA receptors has been explored as a key therapeutic target in pain disorders (Nasstrom et al., 1992; Chapman and Dickenson, 1995; Bannister et al., 2017).

It has been also demonstrated that increased IDO1 activity is inversely related to serotonin concentrations in human plasma (Lood et al., 2015), which has a relevant role in the pain inhibitory descendant modulation (Mico et al., 1997; Sawynok et al., 2001). Sustained Trp catabolism throught KYN pathway during chronic inflammation can compromise the availability of this aromatic amino acid to form serotonin, and thus decrease the serotonin inhibitory descendant pain modulation (Capuron and Dantzer, 2003; Kim et al., 2012). In line with this, a persistent mechanical and thermal hyperalgesia has been shown to be associate with a decreased serotonin/Trp ratio, and an increased KYN/Trp ratio in the hippocampus of rats under chronic arthritis inflammatory pain model induced by a joint CFA injection (Kim et al., 2012).

Clinical and preclinical studies have also demonstrated that the excessive IDO1 activation contributes to inflammation-induced pain (Kim et al., 2012; Huang et al., 2016). IDO1 expression is increased in several inflammatory and pain conditions, for example in the lungs and lymphoid tissue of mice with mechanical pain hypersensitivity induced by an acute and a chronic viral infection, respectively (Huang et al., 2016). In contrast, virus-induced mechanical pain hypersensitivity was not evident in mice lacking IDO1 genes (Huang et al., 2016). In a separate study of chronic arthritis inflammatory pain model, the IDO1 gene expression, protein content and activity were elevated in the hippocampus of rats, resulting in persistent pain hypersensitivity after 21 days of the CFA injection in the tibiotarsal joint (Kim et al., 2012). Blockage of IDO1 with the inhibitor 1-methyl-tryptophan (1-MT) attenuated persistent mechanical and thermal hyperalgesia in rats with chronic arthritis inflammatory pain (Kim et al., 2012). In a clinical observational study, patients affected with chronic back pain showed elevated plasma IDO1 and increased KYN/Trp ratio as compared with healthy controls (Kim et al., 2012).

In murine pre-clinical models, alterations in the KYN pathway and immune system contributing to pain hypersensitivity were demonstrated. For example, in sensory neurons from the DRG and spinal cord, sustained IDO1 and KMO activation due to nerve injury were associated with mechanical and thermal hypersensitivity in rats after 21 the CCI in the sciatic nerve (Rojewska et al., 2016, 2018). Furthermore, in cell culture models we demonstrated that the overexpression of KMO, and subsequent increase in QUIN production is mainly enhanced in monocytic cells, including macrophages and microglia during inflammation (Guillemin et al., 2003). Indeed, the administration of the microglial inhibitor minocycline was able to reduce mechanical hypersensitivity in parallel with a reduction of KMO expression in sensory neurons from rat submitted to the chronic neuropathic pain model induced by the CCI (Rojewska et al., 2014a, 2016). Similarly, treatment with inhibitors for IDO1 (1-MT) or KMO (Ro61-6048 and JM6) attenuated the persistent mechanical and thermal hypersensitivity, along with reduced markers of peripheral inflammation in rat the model of chronic neuropathic pain induced by the sciatic nerve CCI (Rojewska et al., 2016, 2018). These evidence strengthens the correlation between upregulation of KYN pathway, dysregulation of immune system and the development of persistent pain hypersensitivity.

### XA, the Link Between the BH4 Metabolism and the KYN Pathway in Pain

XA was identified as a potent inhibitor of SPR during a screening of a collection of natural compounds (Haruki et al., 2016). This implies that this KYN pathway metabolite has potential to limit the pathological overproduction of BH4 observed in experimental pain models, and therefore induce similar analgesic effects as those elicited by synthetic SPRi, SPRi3 and QM385.

A role for XA in neurotransmission and neuromodulation has been suggested based on (i) the capacity to cross the BBB and spread heterogeneously within different mouse brain regions (Gobaille et al., 2008); (ii) the inhibitory effect on the rat brain vesicular release of glutamate, reducing the glutamatergic transmission (Neale et al., 2013); and (iii) the activity as an agonist of metabotropic glutamate receptors type II, which have been implicated in the negative modulation of nociceptive transmission (Fazio et al., 2016). Thus, the better understanding of the metabolic interaction between these two pathways during inflammation may open new avenues to pharmacologically modulate chronic pain. Indeed, XA has been proposed as an antinociceptive compound, as intraperitoneal administration of XA increased the threshold for nociception in rats (Heyliger et al., 1998) and lower levels of plasma XA were observed in patients affected by episodic and chronic cluster headache in a clinical observational study (Curto et al., 2016). However, to the best of our knowledge, XA-induced decreased SPR activity in a cellular system or a correlation between analgesic effects and SPR inhibition have not been explored yet.

## Conclusion

Advances in the understanding of the mechanisms behind the development of chronic pain have identified a critical interaction between the immune system and the nervous system. These two systems synergistically promote local and systemic responses that restore homeostasis after tissue injury and/or infection. However, this bidirectional communication is also involved in maladaptive feedforward inflammatory loops at multiple levels of the neuroaxis contributing to the development of chronic pain. Neuro-immune interactions are able to control the metabolic flux of various metabolic pathways, including the BH4 and KYN pathways. Both are rapidly activated by inflammation, resulting in the production of several biologically active metabolites, which have been involved in pain states. Recently, XA, a KYN pathway intermediate, was identified as an endogenous inhibitor of the BH4 metabolism. This raises the possibility that XA can potentially modulate the pathological overproduction of BH4 reported in chronic pain hypersensitivity experimental models. The modulation of the KYN pathway can be directed toward the production of XA (through the kynureninase inhibition e.g.), resulting in alterations of BH4. Therefore, understanding the interaction between these two pathways during inflammation is likely to open new avenues to pharmacologically modulate chronic pain.

Altogether, strategies aiming to manipulate the production of bioactive metabolites from the BH4 and KYN pathways, especially in sensory neurons, immune and glia cells might represent promising new analgesic approaches to reduce the hypersensitivity triggered by chronic inflammation. However, given the major central functions of the metabolites produced through the BH4 and KYN pathways in physiological conditions, potential undesirable side effects could be triggered by therapeutic approaches involving the manipulation of both pathways. The relationship between BH4 and KYN pathways, and especially its possible relevance for inflammation-induced pain hypersensitivity, should be critically assessed, and pre-clinical experiments exploring the complex interconnection between both pathways and the production of clinical evidence are encouraged.

## Author Contributions

AS designed the concept and drafted the manuscript. AL, GG, VT, and BH reviewed and edited the manuscript. All authors listed have made a substantial, direct and intellectual contribution to the work, and approved it for publication.

## Conflict of Interest

The authors declare that the research was conducted in the absence of any commercial or financial relationships that could be construed as a potential conflict of interest.

## Abbreviations

1-MT, 1-methyl-tryptophan: an IDO1 inhibitor
3-HAA: 3-hydroxyanthranilic acid
3-HK: 3-hydroxykynurenine
AA: anthranilic acid
AKR: aldoketo reductase
BH4: tetrahydrobiopterin
CAIA: collagen antibody-induced arthritis
CCI: chronic constriction injury
CFA: complete Freund’s adjuvant
CNS: central nervous system
CR: carbonyl reductase
CTLA4: cytotoxic T lymphocyte antigen-4
DHFR: dihydrofolate reductase
DRG: dorsal root ganglia
FDA: Food and Drug Administration
*GCH1*: gene that codes for the human guanosine triphosphate cyclohydrolase I enzyme
*Gch1*: gene that codes for the murine guanosine triphosphate cyclohydrolase I
Glu, glutamate, GTP: guanosine triphosphate
GTPCH: guanosine triphosphate cyclohydrolase I
HIV: human immunodeficiency virus
IASP: International Association for the Study of Pain
IC_50_: concentration at half-maximal inhibition
IDO1: indoleamine 2,3-dioxygenase 1
IFN-γ: interferon gamma
IL-1 β: interleukin-1beta
IL-6: interleukin-6
JM6: a KMO inhibitor
KATs: kynurenine aminotransferases
KMO: kynurenine 3-monooxygenase
KYN: kynurenine
KYNA: kynurenic acid
KYNU: kynureninase
LPS: lipopolysaccharides
NAD^+^: nicotinamide adenine dinucleotide
NMDA: N − methyl − d − aspartate
NMDAR: N-methyl -d-aspartate receptor
NSAIDs: non-steroidal anti-inflammatory drugs
PCD: pterin 4a-carbinolamine dehydratase
PNS: peripheral nervous system
PTPS: 6-pyruvoyl tetrahydropterin synthase
Q-1195: a SPR inhibitor
QM385: a SPR inhibitor
QUIN: quinolinic acid
Ro61-6048: a KMO inhibitor
SNI: spared nerve injury
SPR: sepiapterin reductase
*Spr*: gene that codes for the murine sepiapterin reductase
SPRi: sepiapterin reductase inhibitors
SPRi3: a SPR inhibitor
SSZ: sulfasalazine
TDO: tryptophan 2,3-dioxygenase
TNF-α: tumor necrosis factor alpha
Trp: tryptophan
XA: xanthurenic acid.
